# Screening for *Chlamydia trachomatis* in Low-Risk Obstetric Patients

**DOI:** 10.1155/S1064744994000049

**Published:** 1994

**Authors:** Robert K. Gribble, Jean M. Ricci-Goodman, Richard L. Berg

**Affiliations:** 1 Department of Obstetrics and Gynecology Marshfield Clinic Marshfield WI USA; 2 Department of Medical Biostatistics of the Research Division Marshfield Clinic Marshfield WI USA; 3 Department of Obstetrics and Gynecology Marshfield Clinic 1000 North Oak Avenue Marshfield WI 54449 USA

## Abstract

*Objective:* The purpose of this study was to evaluate the prevalence of *Chlamydia trachomatis* in our rural obstetric population and assess the appropriateness of selective vs. universal prenatal screening.

*Methods:* Between April 1, 1991 and May 1, 1993, 1,587 patients were screened at their first prenatal visit using a *C. trachomatis* antigen test. Patients who were unmarried, younger than 20 years of age, or had a history of a previous sexually transmitted disease (STD) were classified as being at high risk for *C. trachomatis*. All others were considered low risk for *C. trachomatis*.

*Results:* The overall prevalence of *C. trachomatis* was 2.0%. There was a significant difference (*P* < 0.001) in the 1,128 patients considered low risk [0.5%, 95% confidence interval (CI) 0.2–1.2] compared to the 459 patients with one or more identifiable risk factors (5.7%, 95% CI 3.7–8.2).

*Conclusions:* Routine prenatal screening for *C. trachomatis* in our population is not appropriate for low-risk patients.


Infectious Diseases in Obstetrics and Gynecology 1:177–181 (1994)

© 1994 Wiley-Liss, Inc.

### Screening for *Chlamydia trachomatis* in Low-Risk Obstetric Patients

**Robert K. Gribble, Jean M. Ricci-Goodman, and Richard L. Berg**

*Department of Obstetrics and Gynecology (R.K.G., J.M.R.-G.) and Department of Medical Biostatistics of the Research Division (R.L.B.), Marshfield Clinic, Marshfield, WI*

ABSTRACT

***Objective:*** **The purpose of this study was to evaluate the prevalence of *Chlamydia trachomatis* in our rural obstetric population and assess the appropriateness of selective vs. universal prenatal screening.**

***Methods:*** **Between April 1, 1991 and May 1, 1993, 1,587 patients were screened at their first prenatal visit using a *C. trachomatis* antigen test. Patients who were unmarried, younger than 20 years of age, or had a history of a previous sexually transmitted disease (STD) were classified as being at high risk for *C. trachomatis*. All others were considered low risk for *C. trachomatis*.**

***Results:*** **The overall prevalence of *C. trachomatis* was 2.0%. There was a significant difference ($P < 0.001$) in the 1,128 patients considered low risk [0.5%, 95% confidence interval (CI) 0.2–1.2] compared to the 459 patients with one or more identifiable risk factors (5.7%, 95% CI 3.7–8.2).**

***Conclusions:*** **Routine prenatal screening for *C. trachomatis* in our population is not appropriate for low-risk patients.** © 1994 Wiley-Liss, Inc.

KEY WORDS
Pregnancy, infection, prevalence

*Chlamydia trachomatis* is the most common bacterial sexually transmitted disease (STD) in the United States. Many reports have described serious complications that may result from *C. trachomatis* infection during pregnancy. *C. trachomatis* has been implicated in premature rupture of membranes and premature labor and associated with low birth weight, stillbirth, and neonatal death.[1–4] In the postpartum period, *C. trachomatis* has been identified as a cause of late postpartum endometritis.[5] It is important to note, however, that not all studies of *C. trachomatis* in pregnancy have supported the association of *C. trachomatis* with any or all of these complications. Some have found no linkage, while others found an association only in the subset of *C. trachomatis*-infected patients who are also positive for *C. trachomatis* IgM antibodies.[6–11] Less controversial is the neonatal infectious morbidity associated with maternal *C. trachomatis* infection. Of neonates born to mothers with untreated *C. trachomatis*, it is estimated that 30% will develop *C. trachomatis* ophthalmia neonatorum and 35% will develop *Chlamydia* pneumonia.[12] Although these are usually benign and easily treated illnesses, approximately 25% of infants with pneumonia require hospitalization and one report has described permanent respiratory sequelae in infants with pneumonia.[13–14]

The fact that *C. trachomatis* screening of pregnant patients and treatment of those infected can eliminate most or all of the complications described above has led to the recommendation for expanded *C. trachomatis* screening as a worthwhile preventive strategy. The Center for Disease Control (CDC), in a 1985 bulletin, recommended that pregnant women be screened for *C. trachomatis* if

**Address correspondence/reprint requests to Dr. Robert K. Gribble, Department of Obstetrics and Gynecology, Marshfield Clinic, 1000 North Oak Avenue, Marshfield, WI 54449.**

Received September 27, 1993
Accepted January 3, 1994

they were unmarried, younger than 20 years of age, had a history of STDs, or had multiple sexual partners.[15] In 1989, new recommendations were published calling for universal rather than selective prenatal *C. trachomatis* screening.[16] The Wisconsin Department of Health and Social Services and the Wisconsin Association for Perinatal Care have also recommended that all pregnant women be screened for *C. trachomatis.*

Although the cost (and ease) of *C. trachomatis* screening has been reduced by the use of laboratory techniques that do not require cell culture, it is still significant. Furthermore, it has been shown that the prevalence of *C. trachomatis* varies from one geographic area to the next and within geographic areas it varies according to patient risk factors.[1,5,17,18] The purpose of this study was to determine whether it was appropriate, in our particular patient population, to comply with the recommendation to screen all pregnant patients for *C. trachomatis* or if we should continue our policy of selective screening based on certain risk factors.

### SUBJECTS AND METHODS

Between April 1, 1991 and May 1, 1993, all patients who enrolled for routine prenatal care at a large multispecialty clinic in central Wisconsin were considered eligible for this study. Patients who had received *C. trachomatis* screening at other facilities were excluded.

Each patient was interviewed at her first prenatal visit regarding maternal age, marital status, and history of STDs. Patients were classified as being at high risk for *C. trachomatis* if they were younger than 20 years of age, unmarried (single, divorced, separated, or widowed), or had a history of a documented STD. Classification as having had a previous STD required culture-proven gonorrhea, chlamydia, or herpes, a positive serologic test for syphilis or human immunodeficiency virus, or genital condylomata. Patients who had no identified risk factors were defined as being at low risk for *C. trachomatis.*

All enrolled patients, regardless of identified risk factors, were tested for *C. trachomatis* at their first prenatal visit. The Syva MicroTrak™ *Chlamydia* Enzyme Immunoassay (EIA) Specimen Collection Kit (Syra Company, San Jose, CA) was used to obtain and transport the specimen as per the manufacturer's recommendations. Specimens were immediately transported to our in-house regional laboratory which performs *Chlamydia* EIA studies on a daily basis. Specimens are tested according to the Syva Company protocol and utilize the Syva MicroTrak™ II *Chlamydia* EIA kit along with a Denley Wellwash 4 autowasher (Denley Instruments, Sussex, England) and Syva autoreader. Results are reported as either positive or negative for *Chlamydia* antigen.

Patients in the low-risk group who had positive antigen tests were offered confirmatory cell cultures. A specimen from the endocervix was obtained with a sterile swab, placed in a sucrose-phosphate broth, and transported at 4°C to our in-house laboratory. Specimens were inoculated into McCoy cells and incubated for 48–72 h. Monoclonal antibody for *C. trachomatis* was used for staining and the coverslips examined under fluorescent microscopy.

Estimates of the prevalence of *C. trachomatis* are presented by risk group along with exact binomial confidence limits. A comparison of the prevalence for high-risk vs. low-risk patients is based upon an exact chi-square test (CYTEL Software Corporation: Software for Exact Nonparametric Inference User's Manual, Cambridge, MA, 1989). The association of 3 risk factors (marital status, age, and history of STD) with *C. trachomatis* infection was examined through a stepwise logistic regression analysis (SAS Institute, Inc.: SAS/STAT User's Guide, Version 6, Cary, NC, 1989). Results were deemed statistically significant with $P < 0.05$.

### RESULTS

Of the 1,598 eligible prenatal patients, 11 patients did not have *C. trachomatis* studies performed due to patient refusal or the provider not obtaining a specimen. Patient history and *C. trachomatis* test results were available on the remaining 1,587 patients. By history, 1,128 (71%) of these patients were determined to be at low risk for *C. trachomatis* and 459 (29%) at high risk. In the low-risk group, 6 had a positive *C. trachomatis* antigen study, a prevalence of 0.5% [95% confidence interval (CI) 0.2–1.2]. In the high-risk group, 26 had a positive *C. trachomatis* antigen study, a prevalence of 5.7% (95% CI 3.7–8.2). The difference between these 2 groups was highly significant ($P < 0.001$).

Of the 6 patients in the low-risk group who tested positive, 3 agreed to have confirmatory cell

TABLE 1. Prenatal *Chlamydia* infection by risk factors with 95% confidence intervals

| | | History of any STD | | | | | |
|---|---|---|---|---|---|---|---|
| | | No | | | Yes | | |
| Currently married | Age group | Frequency | Percent | 95% CI | Frequency | Percent | 95% CI |
| Yes | 20+ | 6/1,128 | 0.5 | 0.1, 1.0 | 1/93 | 1.1 | 0.0, 5.9 |
| | <20 | 0/23 | 0.0 | 0.0, 14.9 | 0/2 | 0.0 | 0.0, 84.2 |
| No | 20+ | 7/167 | 4.4 | 1.7, 8.5 | 7/52 | 13.5 | 5.6, 25.9 |
| | <20 | 10/114 | 8.8 | 4.3, 15.6 | 1/8 | 12.5 | 0.3, 52.7 |

TABLE 2. Prenatal *Chlamydia* screening: logistic regression analysis

| Risk factor | Odds ratio (95% CI) | *P* |
|---|---|---|
| Unmarried | 7.1 (2.7, 18.9) | < 0.001 |
| Age (years) | 0.89 (0.82, 0.98) | 0.016 |
| History of STD | 2.8 (1.2, 6.5) | 0.017 |

cultures. Two of the 3 cultures were positive for *C. trachomatis*.

Prevalence for each combination of the 3 risk factors is shown in Table 1. Small numbers in some cells were reflected by wide CIs. In particular, although none of the 23 patients whose only risk factor was maternal age younger than 20 years had a positive study, the small number of patients precludes drawing conclusions about this group. A stepwise logistic regression model was used to further assess the relative importance of 3 risk factors. Stepwise logistic regression analysis with maternal age as a continuous variable showed all 3 risk factors to be significant (Table 2).

### DISCUSSION

Several studies have evaluated the question of the cost effectiveness of *C. trachomatis* screening during pregnancy.[12,19,20] Although different methodologies were used, the prevalence of *C. trachomatis* at which screening of a pregnant population became cost effective ranged from 5% to 6%. The major difficulty in determining cost effectiveness is the controversy and uncertainty regarding the importance of *C. trachomatis* in the various pregnancy complications discussed previously. Screening costs and the cost of caring for patients with *C. trachomatis*-related illnesses also vary. With all of this in mind, we believe it is reasonable to assume that *C. trachomatis* screening is not cost effective in a very low-prevalence population such as our low-risk patients. Based on the results of this study, we propose that the vast majority of the benefit of *C. trachomatis* screening will be gained, at a much lower cost, by returning to selective rather than universal screening. This policy is also consistent with a recently published Canadian study.[18]

One of the criticisms of selective screening for *C. trachomatis* is that providers would neglect to do an adequate assessment of risk factors and thus not screen all patients at risk. We decided to simplify risk factor assessment by using only 3 of the 4 risk factors described in the 1985 CDC recommendations. We did not include multiple sexual partners as a risk factor because we were concerned about our ability to objectively assess the accuracy of this history. By limiting risk factors, for the purpose of determining the need to screen for *C. trachomatis*, the process becomes more straightforward and provider compliance will likely improve. We have also subjected the 3 risk factors utilized for this study to logistic regression analysis which documented the importance of each of the 3 risk factors. Finally, we wish to emphasize that this risk factor assessment should not preclude the use of a provider's clinical judgment regarding *C. trachomatis* screening in individual patients.

Another concern about *C. trachomatis* screening is the particular screening test being used. Cell culture remains the "gold standard" but is more costly and associated with greater transport difficulties than are non-culture techniques. Therefore, these non-culture assays have become the most common methods of *C. trachomatis* screening. Although they have excellent sensitivity and specificity, their specificity is still less than that of cell culture.[21] This is not a significant problem in high-prevalence populations, and it is not cost effective to do confirmatory cell cultures for patients in this group who have positive antigen tests. It does, however, become more important in low-prevalence populations. For example, an antigen test with a specificity of 98% and a sensitivity of 90% when applied to a population with a 2.5% disease prevalence has a 54% positive predictive value.[19] This led to our decision to offer confirmatory cell cultures to low-risk patients with positive antigen tests. That 1 of 3 of these confirmatory cultures was negative is consistent with the expected low positive predictive value. The newer DNA polymerase methods are reported to have the same specificity as cell culture and their future use may thus eliminate this concern.[22]

Selective screening requires that one has first determined, as was done in this study, the prevalence in the particular population that is presumed to be low risk. It should not be assumed that prevalences in different populations are comparable. For example, even our high-risk patients had a lower prevalence of *C. trachomatis* than is usually reported for high-risk groups. This may be due to our particular patient demographics, including a rural, central Wisconsin population in which 98% of prenatal patients are Caucasian, 75% have private health insurance, and 85% have at least a high school education. In any case, it emphasizes the need to base screening policies on prevalence measurements from the practitioner's own patient group.

Once it is determined that the population designated as being at low risk does not have a prevalence that justifies, on a cost-effectiveness basis, screening for *C. trachomatis*, the next issue is how one would know if that prevalence changes significantly in the future. One option is to periodically screen low-risk patients until enough have been tested to indicate that the prevalence remains lower than the level at which screening is cost effective. A second option is to rely on statistics generated by the State Health Department (in those states where *C. trachomatis* is a reportable disease), although it is often difficult to draw conclusions about a particular patient population from the more general statistics in these data. We have chosen a third option which is to monitor the prevalence of *C. trachomatis* in the high-risk pregnancy patients and assume that it mirrors the prevalence in low-risk patients. Therefore, if the prevalence increases in high-risk patients, we will need to reevaluate low-risk patients to determine if they have also experienced a concurrent and significant increase. We also monitor the number of positive *C. trachomatis* studies in infants with conjunctivitis or pneumonia, and we would reevaluate the screening protocol if there were more than a minimal number of positive tests (there were none over the time span of this study).

In summary, in an environment of limited health care resources, we believe it is appropriate to use selective rather than universal prenatal screening for *C. trachomatis*. Prerequisites for this approach are a simple method for assessing risk, a knowledge of the *C. trachomatis* prevalence in the particular patient population determined to be low risk, and a mechanism to follow that population for any significant change in prevalence.

### ACKNOWLEDGMENTS

The authors acknowledge the significant assistance of Linda Stini in data collection and coordination.

### REFERENCES

1. Martin DH, Koutsky L, Eschenbach DA, Daling JR, Alexander ER, Benedetti JK, Holmes KK: Prematurity and perinatal mortality in pregnancies complicated by maternal *Chlamydia trachomatis* infections. JAMA 247: 1585–1588, 1982.
2. Gravett MG, Nelson HP, DeRouen T, Critchlow C, Eschenbach DA, Holmes KK: Independent associations of bacterial vaginosis and *Chlamydia trachomatis* infection with adverse pregnancy outcome. JAMA 256:1899–1903, 1986.
3. Alger LS, Lovchik JC, Hebel JR, Blackmon LR, Crenshaw MC: The association of *Chlamydia trachomatis, Neisseria gonorrhoeae*, and group B streptococci with preterm rupture of the membranes and pregnancy outcome. Am J Obstet Gynecol 159:397–404, 1988.
4. Johns Hopkins Study of Cervicitis and Adverse Pregnancy Outcome: Association of *Chlamydia trachomatis* and *Mycoplasma hominis* with intrauterine growth retardation and preterm delivery. Am J Epidemiol 129:1247–1257, 1989.
5. Wager GP, Martin DH, Koutsky L, Eschenbach DA, Daling JR, Chiang WT, Alexander ER, Holmes KK: Puerperal infectious morbidity: Relationship to route of delivery and to antepartum *Chlamydia trachomatis* infection. Am J Obstet Gynecol 138:1028–1033, 1980.
6. Heggie AD, Lumicao GG, Stuart LA, Gyves MT: *Chlamydia trachomatis* infection in mothers and infants. A prospective study. Am J Dis Child 135:507–511, 1981.
7. Hardy PH, Hardy JB, Nell EE, Graham DA, Spence MR, Rosenbaum RC: Prevalence of six sexually transmitted disease agents among pregnant inner-city adolescents and pregnancy outcome. Lancet 2:333–337, 1984.
8. FitzSimmons J, Callahan C, Shanahan B, Jungkind D:

Chlamydial infections in pregnancy. J Reprod Med 31: 19–22, 1986.

9. Harrison HR, Alexander ER, Weinstein L, Lewis M, Nash M, Sim DA: Cervical *Chlamydia trachomatis* and mycoplasmal infections in pregnancy. Epidemiology and outcomes. JAMA 250:1721–1727, 1983.
10. Berman SM, Harrison HR, Boyce WT, Haffner WJ, Lewis M, Arthur JB: Low birth weight, prematurity, and postpartum endometritis. Association with prenatal cervical *Mycoplasma hominis* and *Chlamydia trachomatis* infections. JAMA 257:1189–1194, 1987.
11. Sweet RL, Landers DV, Walker C, Schachter J: *Chlamydia trachomatis* infection and pregnancy outcome. Am J Obstet Gynecol 156:824–833, 1987.
12. Schachter J, Grossman M: Chlamydial infections. Annu Rev Med 32:45–61, 1981.
13. Schachter J, Sweet RL, Grossman M, Landers D, Robbie M, Bishop E: Experience with the routine use of erythromycin for chlamydial infections in pregnancy. N Engl J Med 314:276–279, 1986.
14. Weiss SG, Newcomb RW, Beem MO: Pulmonary assessment of children after chlamydial pneumonia of infancy. J Pediatr 108:659–664, 1986.
15. Anon: *Chlamydia trachomatis* infections. Policy guidelines for prevention and control. MMWR Morb Mortal Wkly Rep 34:53S–75S, 1985.
16. Anon: 1989 sexually transmitted diseases treatment guidelines (published erratum appears in MMWR Morb Mortal Wkly Rep 38:664, 1989). MMWR Morb Mortal Wkly Rep 38:1–43, 1989.
17. Glover DD, Gordon H, Moore G, Larsen B: *Chlamydia trachomatis* antigen prevalence among pregnant women in West Virginia. WV Med J 88:548–551, 1992.
18. Alary M, Joly JR, Moutquin J-M, Labrecque M: Strategy for screening pregnant women for chlamydial infection in a low-prevalence area. Obstet Gynecol 82:399–404, 1993.
19. Carroll JC: *Chlamydia trachomatis* during pregnancy. To screen or not to screen? Can Fam Physician 39:97–102, 1993.
20. Nettleman MD, Bell TA: Cost-effectiveness of prenatal testing for *Chlamydia trachomatis*. Am J Obstet Gynecol 164:1289–1294, 1991.
21. Baselski VS, McNeeley SG, Ryan G, Robison M: A comparison of nonculture-dependent methods for detection of *Chlamydia trachomatis* infections in pregnant women. Obstet Gynecol 70:47–52, 1987.
22. Hosein IK, Kaunitz AM, Craft SJ: Detection of cervical *Chlamydia trachomatis* and *Neisseria gonorrhoeae* with deoxyribonucleic acid probe assays in obstetric patients. Am J Obstet Gynecol 167:588–591, 1992.
